# Pharmacogenomics of drug transporters for antiretroviral long-acting pre-exposure prophylaxis for HIV

**DOI:** 10.3389/fgene.2022.940661

**Published:** 2022-09-29

**Authors:** Nomusa M. Zondo, Parveen Sobia, Aida Sivro, Sinaye Ngcapu, Veron Ramsuran, Derseree Archary

**Affiliations:** ^1^ Centre for the AIDS Programme of Research in South Africa (CAPRISA), Mucosal Immunology Department, Durban, South Africa; ^2^ Department of Medical Microbiology, School of Laboratory Medicine and Medical Sciences, University of KwaZulu-Natal, Durban, South Africa

**Keywords:** African women, PrEP (pre-exposure prophylaxis), female genital tract (FGT), drug transporters, single nucleotid polymorphism (SNP), inflammation

## Abstract

The use of antiretrovirals (ARVs) as oral, topical, or long-acting pre-exposure prophylaxis (PrEP) has emerged as a promising strategy for HIV prevention. Clinical trials testing Truvada^®^ [tenofovir disoproxil fumarate (TDF)/tenofovir (TFV) and emtricitabine (FTC)] as oral or topical PrEP in African women showed mixed results in preventing HIV infections. Since oral and topical PrEP effectiveness is dependent on adequate drug delivery and availability to sites of HIV infection such as the blood and female genital tract (FGT); host biological factors such as drug transporters have been implicated as key regulators of PrEP. Drug transporter expression levels and function have been identified as critical determinants of PrEP efficacy by regulating PrEP pharmacokinetics across various cells and tissues of the blood, renal tissues, FGT mucosal tissues and other immune cells targeted by HIV. In addition, biological factors such as genetic polymorphisms and genital inflammation also influence drug transporter expression levels and functionality. In this review, drug transporters and biological factors modulating drug transporter disposition are used to explain discrepancies observed in PrEP clinical trials. This review also provides insight at a pharmacological level of how these factors further increase the susceptibility of the FGT to HIV infections, subsequently contributing to ineffective PrEP interventions in African women.

## 1 Introduction

HIV remains a formidable public health challenge, with currently over 37.7 million people living with HIV globally (UNAIDS, 2021). At the end of 2020 globally, an estimated 1.5 million new HIV infections were reported, of which 60% occurred in sub-Saharan African (SSA) countries (UNAIDS, 2021). The use and coverage of (antiretrovirals) ARVs has, however, had a positive impact in many SSA countries including South Africa. Despite the advent and use of ARVs, new HIV infections (∼4,000 daily) continue and remain a major concern (UNAIDS, 2021).

The increasing challenge of providing ARVs to a rapidly growing HIV population prompts the need for new interventions to decrease HIV incidence rates (Nicol et al., 2018). Previously tested HIV prevention methods have included the use of ARVs as oral, topical gels or long-acting pre-exposure prophylaxis (PrEP) formulations in uninfected individuals (Abdool Karim et al., 2010; Janes et al., 2018; Nicol et al., 2018). Clinical trials using oral and topical PrEP regimens in high risk heterosexual HIV-serodiscordant couples (Baeten et al., 2012; Thigpen et al., 2012) and men who have sex with men (MSM) (Grant et al., 2010; Molina et al., 2015; Mccormack et al., 2016) reported high levels of protection against HIV acquisition ranging from 44 to 86% (Grant et al., 2010; Thigpen et al., 2012; Mccormack and Dunn, 2015; Molina et al., 2015). However, clinical trials using the same PrEP regimens that focused primarily on at-risk African women, produced inconsistent levels of protection against HIV, ranging from −49 to 39%, with a majority leading to trial termination (Abdool Karim et al., 2010; Van Damme et al., 2012; Marrazzo et al., 2015; Delany-Moretlwe et al., 2018).

The main contributory factor for these low efficacies was identified as low to no adherence to PrEP. However, underlying biological factors beyond adherence have been proposed to play an integral role in low PrEP efficacies (Hu et al., 2015; Nicol et al., 2018). These include drug transporters, which are transmembrane proteins that are expressed ubiquitously in various cells and tissues of the body. Various ARVs used as PrEP have been identified as substrates of different drug transporters (Hu et al., 2015; Taneva et al., 2016; Reznicek et al., 2017). Therefore, drug transporter expression levels and functionality are considered essential for optimal PrEP delivery and for maintaining optimal drug concentrations in cells and tissues targeted by HIV (Hu et al., 2015; Nicol et al., 2018). In addition, there are also host biological factors such as inflammation (Saib and Delavenne, 2021) and genetic polymorphisms (Shenfield, 2004; Arruda et al., 2016) affecting drug transporter disposition, which subsequently affects drug efficacy (Shenfield, 2004; Arruda et al., 2016; Saib and Delavenne, 2021). These findings underscore drug transporters as critical determinants of drug pharmacokinetics. However, there is limited data on drug transporter expression profiles and host factors affecting drug transporter expression and function in anatomical compartments such as the FGT, the predominant site for HIV infection in women during heterosexual intercourse (Hu et al., 2015; Nicol et al., 2018). This warrants the need for further studies that will evaluate these factors, especially in high-risk groups such as African women. The current review, therefore, evaluates various biological factors affecting PrEP pharmacokinetics [absorption, distribution, metabolism and excretion (ADME)] to better understand inconsistencies in PrEP effectiveness observed in clinical trials with at-risk African women.

## 2 Biological, behavioural, and socio-economic factors that increase women’s susceptibility to HIV

Despite noticeable reductions in HIV infections and increases in ARVs accessibility, there are several biological, behavioural and social factors that contribute to higher HIV prevalence rates in women (Ramjee and Daniels, 2013; Abdool Karim et al., 2020). Socio-economic factors that drive high HIV incidence rates in women include sexual abuse, lack of education, lack of food security and the lack of proper social services such as education on HIV and insufficient provision of health services; especially in highly affected regions (Abdool Karim et al., 2012; Ramjee and Daniels, 2013; Nicol et al., 2018; Durevall et al., 2019).

Behavioural factors also play an integral role in high rates of HIV acquisition in young women. These include early age of sexual debut (Mabaso et al., 2018), multiple concurrent sex partners, intergenerational sexual partnering with older men and transactional sexual encounters (Maartens et al., 2014; De Oliveira et al., 2017; Mabaso et al., 2018). Other factors include low marriage rates (Alcaide et al., 2014), intravaginal practices, and low to no condom use due to the inability to negotiate safe sexual practices with their male partners (Ramjee and Daniels, 2013; De Oliveira et al., 2017; Mabaso et al., 2018). Additionally, the use of injectable drugs and alcohol have also been associated with increased HIV transmission through shared needles and high-risk sexual behaviour, respectively (Maartens et al., 2014). Together, these factors suggest that the economic and social disempowerment of young women especially in a developing country such as South Africa contributes largely to high HIV prevalence rates within this population.

Apart from behavioural and socio-economic factors that fuel HIV infections, biological factors also drive higher rates of HIV infections in women. The greater mucosal surface area of the female genital tract (FGT) makes this surface highly susceptible through increased opportunities for target CD4^+^ T cells to become infected with HIV and other sexually transmitted infections (STIs) during sexual intercourse (Ramjee and Daniels, 2013). Other biological factors that increase women’s susceptibility to HIV include bacterial vaginosis (BV) (Heffron et al., 2017; Klatt et al., 2017), vaginal micro-abrasions (Stanley, 2009), cervical ectopy (Critchlow et al., 1995) and genital inflammation (Masson et al., 2015; Mckinnon et al., 2018). Additionally, the use of long-acting injectable progestin hormonal contraceptives (particularly DMPA) has also been associated with increased women’s susceptibility to HIV, however, this remains a topic of ongoing debate, with some studies showing an increased HIV risk (Heffron et al., 2012; Hapgood, 2020) while, others showed no differences (Myer et al., 2007; Shen et al., 2017).

## 3 PrEP in HIV prevention

Since the main route of HIV infection in women is through sexual intercourse, many prevention strategies are aimed at protecting the FGT (Celum, 2011; Nicol et al., 2018). As a result, many different modalities have been tested, which include a vaginal ring containing dapivirine (Baeten et al., 2016), various microbicide gel formulations such as Carraguard (Skoler-Karpoff et al., 2008) and PRO2000 vaginal gels (McCormack et al., 2010) and spermicide gel formulations such as nonoxynol-9 (N-9) (Wilkinson et al., 2002). Besides topical gels and rings, implants containing tenofovir alafenamide (TAF) (Gunawardana et al., 2015) and long-acting injectable formulations containing for example cabotegravir (CAB) (Landovitz et al., 2018) are currently being tested for HIV prevention in PrEP clinical trials. Truvada^®^ which is the co-formulated tenofovir disoproxil fumarate (TDF)/TFV and emtricitabine (FTC)) is a licensed oral PrEP drug that is also used for HIV prevention (Alvarez et al., 2011).

The concept of using TFV as PrEP in preventing HIV infections was initially investigated in macaques with simian immunodeficiency virus (SIV) (Person et al., 2012). During the 1990s, studies on TFV (a nucleotide reverse transcriptase inhibitor of HIV) previously known as (R)-9-(2-phosphonylmethoxypropyl)adenine (PMPA) demonstrated evidence of complete protection against SIV infections (Tsai et al., 1995; Person et al., 2012). The success observed in animal models was, however not fully translated in human HIV prevention clinical trials (Person et al., 2012). One of the major factors that attributed to these inconsistencies was low to no adherence, which limited PrEP exposure to tissues and cells targeted by HIV in areas such as FGT (Rohan and Sassi, 2009; Hu et al., 2015).

## 4 PrEP clinical trials: Efficacy in African women

Significant breakthroughs in using PrEP to prevent HIV infections have been observed in PrEP trials focused-on high-risk HIV-serodiscordant heterosexual couples and MSM (Baeten et al., 2012; Thigpen et al., 2012) (Table 1). The Partners PrEP trial, performed in HIV-serodiscordant heterosexual couples in Kenya and Uganda showed significant HIV reductions of 75 and 67% respectively, with oral TDF-FTC and with TDF alone (Baeten et al., 2012). The TDF2 trial which evaluated the effectiveness of TDF-FTC drugs in sexually active HIV negative heterosexual adults from Botswana; showed that TDF-FTC prevented new HIV infections, by demonstrating a 62% reduction in HIV incidence (Thigpen et al., 2012). In the Pre-exposure Prophylaxis Initiative trial (iPrEX) in MSM from South America, the United States, South Africa and Thailand, a daily single oral dose of Truvada^®^ demonstrated a 44% reduction in HIV incidence (Grant et al., 2010). Similarly, in other MSM European studies testing Truvada^®^ the Pragmatic open-label randomised trial of pre-exposure prophylaxis (PROUD) (Mccormack and Dunn, 2015) and the On-Demand Antiretroviral Pre-exposure Prophylaxis for HIV Infection (IPERGAY) in men who have sex with men (MSM) (Molina et al., 2015), both showed an 86% reduction in HIV incidence (Mccormack and Dunn, 2015; Molina et al., 2015). Results from these studies further supported the effectiveness of PrEP among MSM who are at risk of acquiring HIV (Mccormack and Dunn, 2015; Molina et al., 2015). Currently, in African women, the CAPRISA 004 trial remains the only trial that showed an overall 39% efficacy with a topical gel containing ARV (Table 1) (Abdool Karim et al., 2010). Furthermore, when the data was stratified according to degree of adherence, women with high adherence had a corresponding reduction in HIV incidence by 54%, while women with low adherence had only a 28% reduction in HIV incidence (Abdool Karim et al., 2010).

**TABLE 1:** PrEP clinical trials demonstrating various efficacies in high-risk populations from different regions.

Clinical trials	Study population (regions)	PrEP drugs	PrEP efficacy -reduction in HIV incidence (%)	References
CAPRISA 004	African women (South Africa)	1% TFV gel	39	Abdool Karim et al. (2010)
Partners PrEP	Heterosexual couples (Kenya and Uganda)	Oral TDF-FTC	75	Baeten et al. (2012)
		Oral TDF alone	67	
TDF2	Heterosexual couples (Botswana)	Oral TDF-FTC	62	Thigpen et al. (2012)
iPrEx	MSM (South America, the United States, South Africa, and Thailand)	Oral TDF-FTC	44	Grant et al. (2010)
PROUD	MSM (England)	Oral TDF-FTC	86	McCormack and Dunn, (2015)
IPERGAY	MSM (France and Canada)	Oral TDF-FTC	86	Molina et al. (2015)

Other PrEP clinical trial studies that focused primarily on at-risk African women demonstrated inconsistent levels of protection showing HIV incidence of −49% to 14.5% (Table 2). These include the FEM-PrEP trial (Van Damme et al., 2012) which evaluated, daily Truvada^®^ in women from high-risk areas in South Africa, Kenya and Tanzania. This trial was, however, terminated following low efficacy largely attributed to lack of adherence (Corneli et al., 2016) and low drug concentrations in the FGT (Abdool Karim et al., 2011). The Vaginal and Oral Interventions to Control the Epidemic (VOICE) Microbicide Trial Network (MTN 003) trial was also conducted in women from high HIV prevalence areas in South Africa, Uganda and Zimbabwe (Marrazzo et al., 2015). Women were randomised to either oral TDF, oral TDF-FTC, TFV gel, or respective oral or vaginal placebos. Similar to the FEM-PrEP study, results from this trial showed no efficacy (Marrazzo et al., 2015). The moderate success of the topical CAPRISA 004 1% TFV gel trial led to the Follow on African Consortium for Tenofovir Studies (FACTS-001) (Delany-Moretlwe et al., 2018). The study was conducted at nine community-based clinical trial sites where it assessed the safety and efficacy of the precoitally applied 1% TFV gel in high-risk South African women. Here too, the FACTS-001 trial showed no significant reduction in HIV incidence between the active arm and the control (Delany-Moretlwe et al., 2018).

**TABLE 2:** PrEP clinical trials demonstrating low PrEP efficacies in high-risk populations from different regions.

Clinical trials	Study population (regions)	PrEP drugs	PrEP efficacy -reduction in HIV incidence %	References
FEM-PrEP	African women (South Africa, Kenya and Tanzania)	Oral TDF-FTC	4.7%	Van Damme et al. (2012)
VOICE	African women (South Africa, Uganda and Zimbabwe)	Oral TDF-FTC	−4.4%	Marrazzo et al. (2015)
		Oral TDF alone	−49%	
		1% TFV gel	14.5%	
FACTS-001	African women (South Africa)	1% TFV gel	6.52%	Delany-Moretlwe et al. (2018)

A potential explanation for the disparities in efficacy observed in these PrEP clinical trials could be due to differential drug penetration levels in the rectal compared to the vaginal mucosal tissues (Patterson et al., 2011; Janes et al., 2018). Data from Cottrell et al. (2015), observed that similar levels of adherence of two doses per week of Truvada^®^ reduced HIV incidence by 90% in the MSM population of the iPrEX study, whereas in heterosexual women populations of the FEM-PrEP and VOICE studies low to no protection was observed (Cottrell et al., 2015). Additionally, the complex composition of the vagina’s microbiome and inflammation may affect PrEP disposition in women (Patterson et al., 2011; Klatt et al., 2017; Janes et al., 2018; Mckinnon et al., 2018). These findings urge the need to better understand the mechanisms of drug availability and metabolism within the area of vulnerability, the FGT (Rohan and Sassi, 2009; Hu et al., 2015).

## 5 Compartmental heterogeneity in PrEP drug disposition

The FGT is a highly active and diverse immune environment, with a wide range of heterogeneous immune cells such as macrophages, dendritic cells, Langerhans cells, natural-killer cells, B and T-cells, making it highly susceptible to HIV infections (Hu et al., 2015; Nicol et al., 2018). The increased vulnerability of the FGT to HIV infections is also largely ascribed to the presence of a single columnar epithelium cell layer in the endocervix as opposed to the multi-layered squamous epithelium of the ectocervix (Nicol et al., 2018). These cell layers are vulnerable to micro-abrasions caused by friction during heterosexual intercourse allowing for easy access of HIV (Nicol et al., 2018). A previous study by Shen et al. (2014) also observed that even with intact epithelium, vaginal myeloid dendritic cells expressing HIV receptors can facilitate the capture and dissemination of HIV into the deeper mucosal tissue layers (Shen et al., 2014). The vulnerability of the FGT prompts the need for HIV prevention interventions that provide sufficient protection within all compartments exposed to the virus (Nicol et al., 2018).

Interventions such as PrEP should therefore provide optimal ARV drug concentrations that are sufficient in preventing HIV viral entry, transcription, and replication in HIV targeted cells (Cottrell et al., 2015). Drug exposure of PrEP in the FGT has previously shown variability in drug delivery and availability (Cottrell et al., 2015). To understand this variability; a 14-days open-labelled study by Patterson et al. (2011), demonstrated varying concentration levels of TFV, FTC and their respective active metabolites TFV-DP and FTC triphosphate (FTC-TP) in rectal, vaginal and cervical tissues, following a single dose of Truvada^®^. In rectal tissues, TFV and TFV-DP were detected throughout the 14-day period and concentrations were 100-fold higher when compared to cervical and vaginal tissues; while FTC concentrations were 10 to 15- fold higher in vaginal and cervical tissue when compared to rectal tissue. The active metabolite FTC-TP was, however, only detected for 2 days in all tissues (Patterson et al., 2011). A prior study also compared ARVs drug exposure in cervicovaginal fluid and blood. In the FGT, NRTIs lamivudine (3TC), zidovudine (ZDV), FTC and TFV exhibited high drug concentrations relative to the blood. However, non-nucleoside reverse transcriptase inhibitor (NNRTI) efavirenz (EFV) and protease inhibitors (PIs) lopinavir (LPV) and atazanavir (ATV) exhibited low concentrations in the FGT when compared to the blood (Dumond et al., 2007). These results indicate the heterogeneity of drug disposition and their respective metabolites within sub-compartments of the same anatomical surface or different compartments (Patterson et al., 2011). These studies suggested that certain ARVs may or may not be good PrEP candidates according to their ability to penetrate certain areas (Dumond et al., 2007). In addition, these data further underscore the importance of understanding factors affecting drug pharmacokinetics in tissues that are highly susceptible to HIV infections.

These variations show differential drug penetration levels, giving an insight into the varying levels of protection against HIV observed in some PrEP trials (Cottrell et al., 2015). These discrepancies may be due to the interplay between these PrEP drugs and various membrane-bound proteins that mediate drug transport and availability (Cottrell et al., 2015). For example ARVs such as TFV, FTC and ZDV have been previously shown to be substrates of drug transporters P-glycoprotein (P-pg), multi-drug resistance protein-1 (MRP-1) and organic anion transporters-1 (OAT-1), respectively (Kis et al., 2010; Hu et al., 2015). These data indicated that intracellular and extracellular ARV drug levels can be predominantly regulated by certain drug transporters (Kis et al., 2010; Hu et al., 2015). Therefore, understanding the distribution and biological characteristics of drug transporters may help further define their roles in affecting PrEP efficacy.

## 6 Drug transporters involved in PrEP pharmacokinetics

**FIGURE 1:**
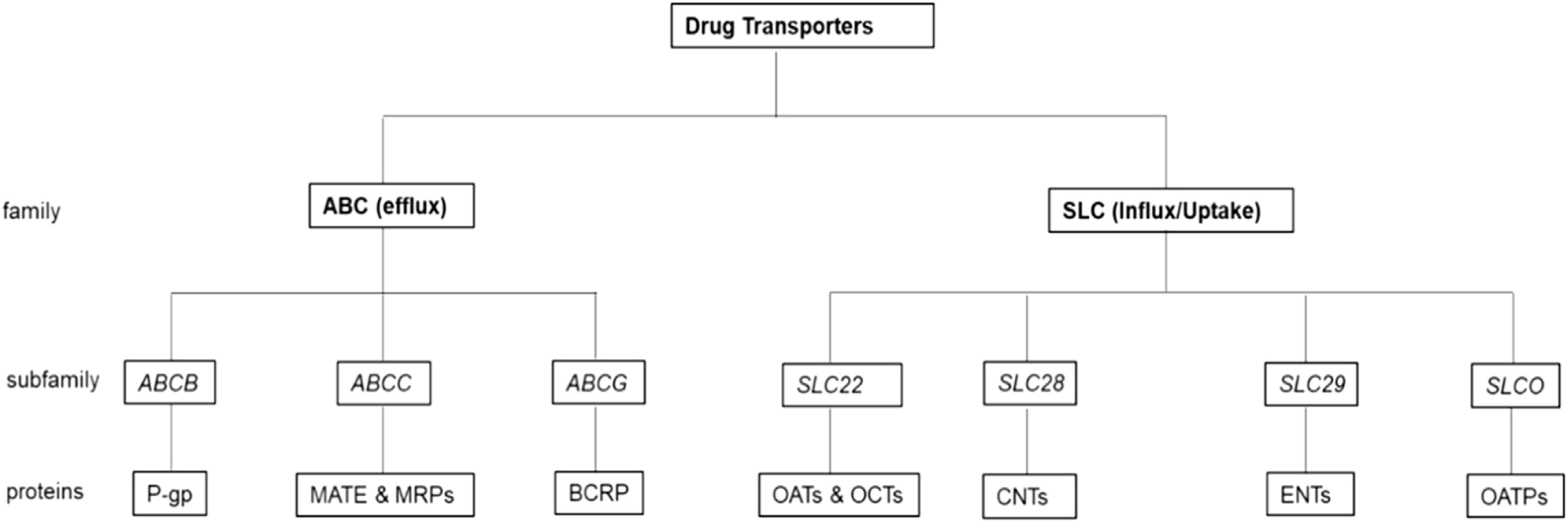
**Drug transporters most relevant in the efflux and influx of PrEP drugs**. In the ABC (efflux) family there are three-drug transporter subfamilies along with their respective proteins that are the most relevant in the efflux of PrEP drugs, while in the SLC (influx/uptake) family, there are four drug transporter subfamilies along with their respective proteins that are the most relevant in the uptake or influx of PrEP.

Drug transporters are types of transmembrane proteins that are ubiquitously expressed in the human body in areas such as the gastrointestinal tract, in epithelial cells in the FGT, lungs, blood-brain barrier, endothelial cells, and liver cells (Zhou et al., 2013; Arruda et al., 2016). Drug transporters comprise of two superfamilies: the ATP-binding cassette (ABC) and Solute Carrier (SLC) proteins (Hu et al., 2015). The ABC proteins are a large family of efflux pumps that bind ATP and utilize its hydrolysis energy to transport molecules across and out of the cell membrane. This family comprises seven subfamilies of which three are the most relevant in the efflux of PrEP drugs (Petzinger and Geyer, 2006; Hu et al., 2015). These include (Figure 1):

i) the *ABCB* subfamily that comprises P-glycoprotein (P-gp),
ii) the *ABCC* subfamily that comprises multidrug and toxin extrusion proteins (MATE) and MRPs, and,
iii) the *ABCG* subfamily that comprises breast cancer resistance protein (BCRP) (Petzinger and Geyer, 2006; Hu et al., 2015; Nicol et al., 2018). The SLC proteins influx or uptake molecules across and into the cell membrane *via* ATP energy dependant carriers or through an electrochemical gradient (Lin et al., 2015). Subfamilies that are the most relevant in the uptake or influx of PrEP drugs include (Figure 1):
i) the *SLC22* subfamily that comprises OAT and organic cation transporters (OCTs),
ii) the *SLC28* subfamily that comprises concentrative nucleoside transporters (CNTs),
iii) the *SLC29* subfamily that comprises equillibrative transporters (ENTs) and
iv) the *SLCO* subfamily that comprises organic anion-transporting polypeptides (OATPs) (Petzinger and Geyer, 2006; Hu et al., 2015; Nicol et al., 2018).

Most of these drug transporters are localised on polarized cells, and regulate substrate distribution on the apical or basolateral surfaces of cells, contributing to the pharmacokinetics of several ARVs (Sissung et al., 2012). In previous studies, TFV and FTC have been shown as substrates of these ABC and SLC drug transporters (Hu et al., 2015; Nicol et al., 2018). This data indicated that the delivery and absorption of these drugs in cells is facilitated by drug transporters, establishing their emerging role as critical determinants in drug pharmacokinetics. This interaction has been noted especially in HIV target cells such as the immune cells of the FGT that include macrophages, vaginal epithelial cells, T cells, and dendritic cells expressing CD4 receptors (Hu et al., 2015; Taneva et al., 2016), and also in peripheral blood mononuclear cells (PBMCs) and epithelial cells of the intestinal and renal system (Kerb, 2006).

### 6.1 Role of drug transporters in the FGT

The extracellular accumulation of TFV and FTC in cells overexpressing certain drug transporters has been demonstrated in various studies focused on the FGT. Findings from these studies suggested that the delivery of effective PrEP drug concentrations to cells and tissues in the FGT is highly associated with the mRNA expression level and functionality of drug transporters (Grammen et al., 2014; Nicol et al., 2014; Hijazi et al., 2015; Taneva et al., 2016). Zhou et al. (2013) showed varying drug transporter expression levels in the FGT (vaginal and ectocervix tissues) and liver. mRNA expression was defined as ≤2% (undetectable), 2%–10% (low expression), 10%–50% (moderate expression) and 50%–100% (high expression) (Zhou et al., 2013). In the FGT, high mRNA expression of drug transporters (MRP-1, MRP-4, P-gp, BCRP, ENT-1 and OCT-2) was observed when compared to the liver, while MRP-2 and influx drug transporters OAT-1 and OAT-3 showed moderate and low mRNA expression as compared to the liver (Zhou et al., 2013). Similarly, a study by Taneva et al. (2016) also showed significantly low expression of uptake drug transporters OAT-1 and OAT-3 in vaginal epithelial cells and T-cells, which accounted for the poor permeability of TFV across the cell membranes and into the cells. Additionally, this study showed that the *in-vitro* transfection of T cells with the drug transporter OAT-1 increased TFV uptake, resulting in high intracellular drug accumulation (Taneva et al., 2016). These studies indicated that there is variability in drug transporter expression levels within different tissues. Therefore, analysing the expression levels of drug transporters in the FGT could aid in better understanding their role in the pharmacokinetics of drugs (Zhou et al., 2013; Taneva et al., 2016).

Nicol et al. (2014) showed high mRNA expression levels of efflux drug transporters P-gp and MRP-2 in vaginal tissues compared to colorectal tissue, while MRP-4 was only highly expressed in colorectal tissues. In contrast, uptake drug transporters OAT-1, OAT-3 and OATP1B1 exhibited extremely low to no expression in colorectal and vaginal tissues, respectively (Nicol et al., 2014). Additionally, immunohistochemistry that informed on the localisation of these drug transporters revealed high protein expression of P-gp and MRP-2 in vaginal epithelial cells compared to colorectal epithelial cells, while low to no protein expression of OAT-1 was observed in colorectal epithelial and vaginal cells, respectively (Nicol et al., 2014). Differences in protein localisation and expression suggested an increased expression of efflux drug transporters in vaginal tissues compared to colorectal tissues (Nicol et al., 2014). These data show that more drug is pumped out of cells in the vagina, while an increased expression of uptake drug transporters in colorectal tissues promoted an uptake of drugs (Nicol et al., 2014). These findings highlighted that inter-tissue variability in drug transporter expression may contribute to the greater intracellular accumulation of ARVs such as TFV and maraviroc in colorectal tissues compared to vaginal tissues (Nicol et al., 2014). High expression levels of efflux drug transporters P-gp, MRP-2 and BCRP in vaginal and endocervical tissues was also reported by Grammen et al. (2014). The study further established in intestinal cell lines-Caco-2 and vaginal epithelial cell lines-SiHa using specific drug transporter inhibitors, that ARV drugs darunavir, maraviroc and saquinavir are substrates of efflux drug transporters P-gp and MRP-2, which are likely to contribute to lower intracellular levels of these respective drugs (Grammen et al., 2014).

To further understand the role of drug transporters in the mucosal compartment, the relationship between the accumulation of topically applied PrEP drugs dapivirine, darunavir and TFV, and the expression of drug transporters was characterised in cervicovaginal cell lines (Hijazi et al., 2015). These included HeLa cell lines, VK2/E6E7, Ect1/E6E7 and End1/E6E7 derived from human cervical epithelial adenocarcinoma, primary vaginal, ectocervical and endocervical epithelial cells, respectively (Hijazi et al., 2015). Tenofovir significantly downregulated the mRNA expression of MPR5 in VK2/E6E7, while dapivirine significantly upregulated most MRP drug transporters in all cell lines. Darunavir stimulation also significantly upregulated the uptake drug transporter CNT3 in all cells, while MRP3 was only significantly unregulated in VK2/E6E7 cell line (Hijazi et al., 2015). This characterisation by Hijazi et al. (2015) provided insight not only on the type of drug transporters present in the FGT but also how drug transporter disposition may be altered by the presence of certain drugs; which could assist in the assessment of ARV pharmacokinetics in the FGT. Furthermore, these findings could assist in the determination of suitable PrEP drug formulations that could provide sufficient drug concentrations to susceptible tissues and cells of the FGT (Hijazi et al., 2015).

### 6.2 Role of drug transporters in peripheral blood mononuclear cells

A study by Turriziani et al. (2008) determined the mRNA expression levels of drug transporters in PBMCs (isolated from buffy coats) from HIV infected individuals failing ARV therapy and HIV negative individuals. The mRNA expression levels of P-gp, MRP (-1,-4 and -5) was significantly higher in HIV infected individuals compared to HIV negative individuals (Turriziani et al., 2008). A higher inter-individual mRNA expression variability was also observed in HIV infected individuals, indicating a correlation between the presence of ARVs and drug transporter expression levels (Turriziani et al., 2008). Similarly, Bousquet et al. (2009) investigated if the singular or combined (dual or triple) use of TFV, FTC and EFV on PBMCs isolated from healthy donors disrupts mRNA drug transporter expression levels (Bousquet et al., 2009). Following a 20-h *in-vitro* incubation, a singular use of FTC induced MRP5, while TFV reduced MRP (-1,-5,-6) and P-gp mRNA expression in PBMCs (Bousquet et al., 2009). FTC was also shown to exhibit an inhibitory effect on the mRNA expression of efflux drug transporter MRP-1 in a dose-responsive manner. These findings suggest a correlation between the presence of FTC with MRP-1 expression (Bousquet et al., 2008). The use of ZDV was also previously shown to be associated with the upregulation of efflux drug transporters MRP-1 and MRP5 expressed on PBMCs (Jorajuria et al., 2004). Findings from these studies showed that an interaction between ARVs and drug transporters may alter drug transporter disposition by affecting mRNA expression levels; subsequently affecting intracellular drug accumulation (Jorajuria et al., 2004; Bousquet et al., 2009).

Contrary to these studies, Falasca et al. (2011) and Giraud et al. (2010) showed no correlations between the mRNA expression levels of efflux drug transporters and the presence of ARVs ritonavir (RTV), ATV, and LPV in PBMCs isolated from HIV infected patients (Giraud et al., 2010; Falasca et al., 2011). The study found no variation in the mRNA expression levels of P-gp and MRP drug transporters before and after ARV intake (Falasca et al., 2011). However, in a more recent study, a significant association was observed between ARVs and drug transporters P-gp, BCRP, MRP-1, ENT-2 and OCT-1 expressed on monocytes and monocyte-derived macrophages isolated from HIV negative individuals and HIV infected individuals receiving ARV therapy containing either abacavir, ATV, EFV, rilpivirine, TFV, 3TC, FTC, elvitegravir, dolutegravir, and cobicistat (Hoque et al., 2021). These findings showed that these associations could lead to sub-optimal intracellular drug concentrations, subsequently allowing HIV infections in HIV negative individuals or further HIV replication in HIV infected individuals (Hoque et al., 2021).

### 6.3 Role of drug transporters in the renal system

The entry of TFV into epithelial cells of the kidney tubule is mediated by the uptake drug transporters OAT-1 and OAT-3 expressed on its basolateral membrane (Cihlar et al., 2007), while the efflux of TFV into urine is mediated by efflux drug transporter MRP-4 expressed at the apical side of renal proximal tubules (Ray et al., 2006). These data together provide evidence that TFV is a substrate of OAT-1, OAT-3 and MRP-4 drug transporters expressed in renal tubules (Ray et al., 2006; Cihlar et al., 2007). TFV is also a substrate of the efflux drug transporter MRP-8 expressed in renal proximal tubules since higher cytotoxic concentrations of the drug were observed in cells overexpressing MRP-8 (Tun-Yhong et al., 2017). The uptake of ARVs cidofovir, adefovir and TFV was evaluated in human embryonic kidney (HEK293) cells transfected with uptake drug transporters OCT-2, OAT-1 and OAT-3 (Uwai et al., 2007). Results showed higher uptake of all ARVs through OAT-1 compared to OAT-3, while OCT-2 exhibited no uptake, indicating that OAT-1 plays a significant role in renal transport of these ARVs (Uwai et al., 2007). Similarly, renal secretion of FTC was mediated by MATE-1 which functionally acts as an efflux drug transporter, expressed on the apical side of renal proximal tubules (Reznicek et al., 2017).

These studies collectively provide insight that ARV drug levels are not only determined by drug adherence but also by other factors such as the presence of specific drug transporters and their expression levels. However, definitive conclusions on the full effects of drug transporters on ARV pharmacokinetics in at-risk groups such as young women especially in Africa have not been drawn. The paucity of data on African women warrants the need for new studies to fully understand:

i) the effect of ARVs on drug transporters expression,
ii) how varying drug transporter expression levels influence ARV penetration in vulnerable areas such as the FGT, and
iii) how different biological factors such as inflammation and polymorphisms may also affect drug transporter expression and function.

## 7 Biological factors modulating drug transporter expression and function

### 7.1 Genetic polymorphisms

Pharmacogenetic research has been used as a tool to determine individuals’ susceptibility to certain diseases and for the customisation of drug therapies according to patient’s genetic blueprint (Sissung et al., 2012; Castellanos-Rubio and Ghosh, 2019). As such, sequencing and genotyping technology have been widely used to identify and determine the effect of variants such as genetic polymorphisms in various genes. There are four types of genetic polymorphisms that have been shown to regulate genes (Ismail and Essawi, 2012). These include:

I. small insertions and deletions (InDels) which is a deletion or insertion in the DNA sequence (Boschiero et al., 2015),
II. interspaced or tandem repeat polymorphisms which are tandemly repeated nucleotides of approximately ≥2 base pairs (bp) in DNA sequences (Ismail and Essawi, 2012),
III. structure or copy-number variations (CNVs), polymorphisms which are various copies of differently sized segments of nucleotides in DNA sequences (Stankiewicz and Lupski, 2010) and
IV. single nucleotide polymorphisms (SNPs), which are point mutations of nucleotide bases within DNA sequences (Sissung et al., 2012; Yee et al., 2018).

Types of SNP variations include missense mutations or nonsynonymous substitutions which is a single nucleotide change within a codon, subsequently resulting in the coding of a different amino acid (Hunt et al., 2009). The presence of such mutations on protein binding sites may affect substrate binding, while those not found on protein binding sites may affect protein expression levels. For example, the missense mutation rs2273697 located on the efflux drug transporter gene *ABCC2* encoding MRP-2 results in a change from valine to isoleucine, on exon 417 (V417I), affecting its expression levels (Yee et al., 2018). Another type of SNP mutation is a silent mutation or synonymous substitutions, which are single nucleotide point mutations on a codon that do not result in an amino acid change (Hunt et al., 2009). However, these may still affect RNA transcription and stability that may affect mRNA expression levels and protein binding (Yee et al., 2018). For example, the SNP rs1045642 located on the efflux drug transporter *ABCB1* gene (3435C/T Ile1145Ile) encoding P-gp is a type of silent mutation that has been highly studied in drug pharmacokinetics (Sissung et al., 2012; Yee et al., 2018). This SNP has been also previously associated with low P-gp expression levels in the duodenum which correlated with an increase of digoxin plasma concentrations (Hoffmeyer et al., 2000). The presence of certain genetic variations in drug transporter genes has sparked a huge interest in further understanding their functional effect; especially since SNPs in certain drug transporter genes have been shown to modulate their function by affecting protein folding, expression levels, and their ability to bind substrates and regulate drug pharmacokinetics (Sissung et al., 2012; Yee et al., 2018).

SNPs involved in the pharmacokinetics of ARVs have led to adverse effects and varied ARV therapy outcomes amongst HIV infected patients (Shenfield, 2004; Arruda et al., 2016). Arruda et al. (2016) showed the association between SNPs in drug transporter genes and intolerance to ARVs in a cohort of HIV infected Brazilian participants (Arruda et al., 2016). Results showed an association between variations in *ABCC2* genes (rs3740066 and rs4148396) encoding MRP-2 and intolerance in patients taking regimens containing either LPV, RTV, indinavir or ATV PIs; while variations in *SLCO2B1* genes (rs2712816, rs12422149, rs1676885 and rs949069) encoding OATP2B1 caused intolerance in patients taking regimens containing stavudine or ZDV nucleotide reverse transcriptase inhibitors (NRTIs) (Arruda et al., 2016). The presence of the C allele on the *ABCC1* gene 198217C/T (rs212091) encoding MRP-1 and the TT genotype on the *ABCB1* gene 3435C/T (rs1045642) encoding P-gp; was also shown to be possibly associated with reduced gene expression in an HIV infected Brazilian participants receiving highly active antiretroviral therapy (HAART) regimens; subsequently affecting the efflux of ARV regimens containing PIs, leading to an increased risk of virological failure (Table 3) (Coelho et al., 2013).

**TABLE 3:** Effects of SNPs in drug transporter genes involved in the pharmacokinetics of ARVs in different ethnic groups.

SNPs	Ethnic group	ARVs	SNPs effect	Genotype causing effect	Genotype frequency Number of patients n (%)	References
*ABCC2* 224C/T (rs717620)	Thailand	TFV, Lamivudine, Efavirenz	Increased TFV plasma concentration	CC	CC 67 (57); CT 45 (39); TT 5 (4) n = 117	Manosuthi et al. (2014)
	Japanese	TFV, Emtricitabine, Darunavir, Ritonavir	TFV induced-KTD	CC	CC 18 (94.7); CT 1 (5.3); TT 0 (0) n = 19	Nishijima et al. (2012)
	Caucasian	TFV	TFV induced-KTD	CC	CC 9 (60.0); TT 1 (6.7); CT 5 (33.3) n = 15	Danjuma et al. (2018)
*ABCC4* 4131T/G (rs3742106)	Thailand	TFV	Increased TFV plasma concentration	TG/GG	TT 34 (22.7); TG 80 (53.3); GG 36 (24.0) n = 150	Rungtivasuwan et al. (2015)
*ABCC1* 198217C/T (rs212091)	Brazilian	Zidovudine, Lamivudine, Efavirenz/Nevirapine; Lopinavir/Ritonavir	Increased risk of virological failure	CC	CC 62 (84.9); TC 10 (13.7); CC 1 (1.4) n = 73	Coelho et al. (2013)
*ABCB1* 3435C/T (rs1045642)				TT	CC 37 (50.7); CT 25 (34.2); TT 11 (15.1) n = 73	
*ABCC4* 4976C/T (rs1059751)	Thailand	TFV	TFV induced-KTD	CC	CC 20 (37.0); TT 9 (16.7); CT 25 (46.3) n = 54	Likanonsakul et al. (2016)
*ABCC4* 3436A/G (rs1751034)	Caucasian	TFV	TFV induced-KTD	GG	AA 27 (64.3.); AG 9 (21.4); GG 6 (14.3) n = 42	Salvaggio et al. (2017)
*SLCO1B1* 463C/A (rs11045819)	African	Rifampin	Low plasma concentrations	CC	CC 30 (81); CA 7 (19) n = 37	Weiner et al. (2010)
	Ghanaian	Rifampin	High plasma concentrations	CC	CC 95 (84.1); CA 17 (15); AA 1 (0.09) n = 113	Dompreh et al. (2018)

Fellay et al. (2002) showed that the TT genotype on the *ABCB1* gene 3435C/T in an HIV infected Caucasian population was associated with low P-gp expression in PBMCs, affecting ARV concentrations (Fellay et al., 2002), However, a subsequent study showed that virological failure was associated with the CC genotype of the *ABCB1* gene 3435C/T instead of the TT genotype in HIV infected patients from the province of British Columbia in Canada (Brumme et al., 2003). To elucidate variations of the *ABCB1* 3435C/T SNP observed in these studies; prior results by Ameyaw et al. (2001) that assessed the frequency of this SNP in ten ethnic groups can be used (Ameyaw et al., 2001). Results showed noticeable differences in the SNP frequencies between African, Asian and European populations. The C allele was highly present in the African populations compared to Asian and European populations which exhibited high frequencies for the CT and TT genotypes (Ameyaw et al., 2001). Schaeffeler et al. (2001) also supported these findings by reporting a high frequency of the CC genotype in the *ABCB1* gene 3435C/T of West African and African American populations compared to the T allele (Schaeffeler et al., 2001). These findings could imply possible variations in drug transporter genes which could lead to varied ARV therapy outcomes in the African vs. Caucasian populations (Ameyaw et al., 2001; Schaeffeler et al., 2001).

Pharmacogenetic studies conducted with African populations have also shown high genetic diversity, which subsequently leads to varied drug transporter function and expression levels; impacting drug pharmacokinetics differently as reviewed by Rajman et al. (2020). The presence of the *SLCO1B1* SNP 463C/A rs11045819 encoding the OATP1B1 protein was shown to impact rifampin pharmacokinetics differently in African populations (Weiner et al., 2010; Dompreh et al., 2018). A study by Weiner et al. (2010) showed that a high frequency of the CC genotype for the *SLCO1B1* 463C/A (rs11045819) gene was associated with low rifampin concentrations in African individuals during multidrug intensive therapy against TB (Weiner et al., 2010). However, in an African Ghanaian population also exhibiting a high frequency CC genotyping for the same gene taking standard first-line TB therapy; no effect on rifampin was observed (Dompreh et al., 2018) Table 3. Similarly studies by Chigutsa et al. (2011) and Gengiah et al. (2014) on the *SLCO1B1* (rs4149032) SNP both reported an associated between high SNP frequency and low rifampin plasma concentrations in TB and HIV-TB co-infected South African individuals taking rifampin (Chigutsa et al., 2011; Gengiah et al., 2014). This association was however, not observed in a TB infected Ghanaian population taking standard first-line TB therapy containing rifampin which also exhibited a high frequency for this SNP (Dompreh et al., 2018). The effect of the *ABCB1* SNP 4036G/G (rs3842) encoding P-gp on efavirenz was evaluated in different African populations. In an HIV infected South African population the AG and GG genotypes were significantly associated with decreased efavirenz plasma concentrations (Swart et al., 2012), however the GG genotypes in a healthy Ugandan population was associated with higher efavirenz plasma concentrations (Mukonzo et al., 2009). Similarly in Ethiopian and Tanzanian HIV infected populations the presence of the G allele was associated with higher efavirenz plasma concentrations, with higher frequency of the G allele observed in Tanzanians (Ngaimisi et al., 2013). These data indicated that the effects of SNPs may differ among African populations; therefore, in order to make definitive conclusions that a SNP affects the African population in a certain way, the functional or expressional effect SNPs should be tested among a wide range of different African populations as reviewed by Rajman et al. (2020). Despite the small sample size and sparsity of these data in various studies with African populations as reviewed in Rajman et al. (2020), these data do add to the understanding of how SNPs can impact drug pharmacokinetics in the African population. Together these studies could be used to adjust the standard recommended dose of ARV and TB drug for the African population that accounts for the presence of SNPs (Dandara et al., 2011; Rajman et al., 2020).

The effects of SNPs on drug transporter genes have also been associated with increased plasma concentrations of TFV. Studies on an HIV-infected cohort in Thailand showed higher TFV plasma concentrations in patients with the CC genotype on the *ABCC2* 224C/T gene (rs717620) encoding MRP-2 compared to patients with the *ABCC2* TT or CT genotypes (Table 3) (Manosuthi et al., 2014). Similarly, another Thailand study by Rungtivasuwan et al. (2015) reported higher TFV plasma concentrations in HIV infected patients with the *ABCC4* 4131 (rs3742106) TG or GG genotypes (encoding MRP-4) compared to patients with the *ABCC4* TT genotype (Table 3) (Rungtivasuwan et al., 2015). These studies proposed that polymorphisms in these drug transporter genes may alter their gene expression or function in renal tubules leading to more effluxed drug and reduced glomerular filtration which is involved in TFV renal clearance; resulting in higher plasma concentrations (Manosuthi et al., 2014; Rungtivasuwan et al., 2015). A more recent study also showed in an HIV infected Caucasian population a significant association of the CC genotype in the *ABCC2* 224C/T gene with high TFV plasma concentrations, resulting in an increased risk of TFV induced-kidney tubular dysfunction (KTD) (Table 3) (Danjuma et al., 2018). Nishijima et al. (2012) previously confirmed that the CC genotype in the *ABCC2* 224C/T gene leads to high TFV plasma concentrations resulting in the induction of KTD or renal toxicity in Japanese patients (Table 3) (Nishijima et al., 2012). While the presence of the TT genotype in the *ABCC4* 4131T/G gene was not associated with TFV induced-KTD, the study attributed these findings to inter-individual variability in genetic backgrounds, which may cause patients to respond differently to the same drug (Kerb, 2006; Nishijima et al., 2012). Other SNPs on the *ABCC4* gene that have been associated with increased plasma TFV concentrations were evaluated in two studies; in an infected population from Thailand with the C allele on the ABCC4 4976C/T gene (Likanonsakul et al., 2016), and in a Caucasian population with GG genotype on the *ABCC4* 3436A/G gene (Salvaggio et al., 2017).

Reports obtained from these studies highlight the importance of understanding how the presence of SNPs may affect the efficacy of ARVs by affecting drug transporters’ expression and function. Furthermore, these findings could be used to identify populations who are at a higher risk of developing adverse effects due to the presence of certain SNPs. However, most of these studies on SNPs in drug transporter genes affecting ARVs have been performed in non-African populations. Since SNP frequency differs significantly among different ethnic populations, more comprehensive investigations of SNPs in drug transporter genes are required, especially in the populations of African ethnicity. Data from populations of African descent will help us better understand how genetic diversity within these populations and SNPs influence drug transporter genes and subsequently lead to effective or ineffective therapy.

Pharmacogenetic research on polymorphisms present in drug transporter and drug-metabolizing genes is also vital in precision medicine, which enables the tailoring of effective therapies based on patients’ genetic backgrounds (Hockings et al., 2020). The advantage of a precision medicine approach is the ability to predict putative ineffective therapies and possibly reduce adverse reactions (Hockings et al., 2020). Since there are reports of increased adverse reactions in patients in populations of African ethnicity taking ARVs, precision medicine is highly important in HIV prevention and treatment (Hockings et al., 2020). Patients taking ARV regimens containing EFV in SSA were predisposed to EFV-induced neuropsychiatric adverse reactions, due to specific genetic variants that reduced the functionality of cytochrome P450 2B6 (*CYP2B6*) the enzyme involved in EFV metabolism (Masimirembwa et al., 2016). One of the genetic variants of *CYP2B6* 516G>T (rs3745274) reported a frequency of 34%–50% in African populations compared to 15 and 20% in white populations (Masimirembwa et al., 2016). However, when the EFV dosages in ARV regimens were further titrated and reduced, there was improved EFV metabolism leading to significantly reduced neuropsychiatric adverse reactions (Gatanaga et al., 2007; Masimirembwa et al., 2016). These disparities in frequencies between the populations could lead to varied enzyme metabolism when similar drugs are used which may lead to ineffective drug metabolism and availability. This data highlights the importance of using pharmacogenetic research in guiding the development of precision medicine, especially in highly affected populations ensuring effective drug dosing, delivery, and metabolism.

### 7.2 Genital inflammation

Genital tract inflammation has been identified as an elevated profile of five of any of the nine pro-inflammatory cytokines (MIP-1α, MIP-1β, IP-10, IL-8, MCP-1, IL-1α, IL-1β, IL-6, and TNF-α) above the 75th percentile for each cytokine in a previous CAPRISA study (Abdool Karim et al., 2010; Masson et al., 2015) Genital inflammation creates an environment conducive for HIV infection and replication (McKinnon et al., 2018) increasing the risk for HIV by more than three-fold (Masson et al., 2015). The role of genital inflammation in undermining PrEP efficacy was demonstrated in a study by McKinnon et al. (2018). A 57% protective efficacy was found in women with no genital inflammation compared to 3% in women with genital inflammation (McKinnon et al., 2018). Although the mechanisms to explain why some people have comparatively high levels of genital inflammation while others do not are not fully understood, a likely driver of genital inflammation is bacterial vaginosis (BV), a microbial dysbiosis common in reproductively active women (Klatt et al., 2017). BV also plays a role in significantly modifying PrEP efficacy (Klatt et al., 2017). Klatt et al. (2017) showed that TFV gel reduced HIV incidence by 61% in women with a *Lactobacillus* dominant vaginal microbiome compared to only 18% in women with a *non-Lactobacillus* dominant vaginal microbiome (Klatt et al., 2017). Furthermore, sexually transmitted infections (STIs) (Masson et al., 2014) and exogenous hormonal contraceptives (HCs) (Deese et al., 2015) are also significantly associated with genital inflammation, through the secretion of pro-inflammatory cytokines (Masson et al., 2014; Deese et al., 2015). The mechanisms by which all these factors individually or collectively interplay with drug transporter disposition, drug levels and in turn PrEP efficacy remains less well defined.

#### 7.2.1 Role of inflammation-induced cytokines, in modulating drug transporter expression and function

The impact of inflammation on drug transporter expression and function has been examined in tissues of the intestines, kidneys, and blood-brain barrier (Petrovic et al., 2007; Saib and Delavenne, 2021). Despite the lack of data regarding the direct mechanisms involved; inflammation-mediated changes in drug transporter expression and function have been implicated in significantly impacting drug pharmacokinetics (Petrovic et al., 2007; Cressman et al., 2012). In an *in vitro* study, human brain cell lines (hCMEC/D3) treated with IL-6 and IL-1β, resulted in the downregulation of BCRP and P-gp expression levels (Poller et al., 2010). Additionally, the induction of IL-6 and IFN-γ on primary human hepatocytes was also shown to downregulate the mRNA expression levels of the efflux drug transporters BCRP, MRP-2, and MRP-3 and influx/uptake drug transporters OATP (-2B1,-1B1,-1B3) (Le Vee et al., 2009; Le Vee et al., 2011). Previous studies corroborated similar findings of inflammation IL-6 induced downregulation of P-gp expression on rat hepatocytes and human hepatoma cell lines (Sukhai et al., 2000). Similarly human cell line Caco-2 pre-treated with TNF-α significantly decreased intestinal P-gp expression, while IFN-γ had no effect (Belliard et al., 2004). In rats with endotoxemia, high levels of IL-6 and IL-1β reduced the mRNA expression levels of P-gp and MRP-2 in intestinal tissues (Arana et al., 2017). This lipopolysaccharide-induced endotoxemia in rats model showed that there was IL-1β induced downregulation of MRP-2 in enterocytes (Arana et al., 2020). These various cellular and small animal models demonstrate how infection and inflammation-induced cytokines can modulate drug transporter disposition. The caveat to the methods used in these models is that mRNA expression levels may not directly reflect functional proteins expressed. Future investigations are therefore required and should include both mRNA expression to its corresponding protein. There are also other biological factors related to inflammation that could also affect drug transporter disposition.

### 7.3 Role of toll-like receptors and pH in modulating drug transporter expression and function

#### 7.3.1 Toll-like receptors-induced inflammation

TLRs are pattern recognition receptors, these receptors recognise pathogen-associated molecular patterns located on various microbes for example Pam3CSK-4 and lipopolysaccharide (LPS) which are TLR-2 and TLR-4 agonists, respectively (Cario, 2016; Suzuki et al., 2017). TLRs are activated by the binding to their respective agonists. This interaction causes the stimulation of appropriate signalling pathways in innate and adaptive immune cells which then regulate drug transporter expression levels (Cario, 2016; Suzuki et al., 2017). These TLR-mediated changes in drug transporter expression have been evaluated in the progression of atherosclerosis. To determine which downstream transcriptional signalling pathways were involved in this interaction; *in-vitro* testing using mouse macrophage cell lines (Raw 264.7) stimulated with TLR-2 and TLR-4 agonists Pam3CSK-4 and Lipid-A, respectively; were performed (Suzuki et al., 2017). Expression of myeloid differentiation primary-response protein 88 (MyD88), Toll/interleukin-1-domain-containing adapter-inducing interferon β (TRIF), liver X receptors (LXR), interferon regulatory factor 3 (IRF3), and the phosphorylation of nuclear factor kappa B (NF-kb) were determined with TLR-2 and TLR-4 activation. These results showed a differential pattern of significantly increased MyD88, LXR and NF-kb expression and low TRIF and IRF3 expression (Suzuki et al., 2017). This coincided with the significant upregulation of *ABCA1* expression levels, while ABCG1 expression levels were downregulated. TLR-2 stimulated cells pre-treated with NF-kb and p38 inhibitors MG-132 and SB203580, respectively suppressed the expression of *ABCA1*. These data provided evidence of the sensitivity of drug transporter expression to signal transduction–the MyD88, LXR, NF-kb and p38 pathways. These data provide support to the hypothesis that inflammation modulates the expression of drug transporters which can then lead to disease pathogenesis (Suzuki et al., 2017).

#### 7.3.2 Sensitivity of drug transporter function to pH

The level of acidity or alkalinity (pH) in extracellular fluids is an additional factor that has been shown to modulate drug transporter function (Breedveld et al., 2007). The function of the efflux drug transporter BCRP was determined in Madin-Darby canine kidney (MDCK II) cells grown in pH adjusted media and exposed to methotrexate (MTX). At acidic pH levels, the efflux transporter BCRP pumped out MTX more efficiently when compared to physiological and basic pH levels. This data highlighted the possible clinical implications that the function of BCRP is pH-sensitive in the extracellular environment, thereby affecting intracellular concentrations and the effectiveness of MTX (Breedveld et al., 2007). These data suggest that pH is an additional factor that can also modulate drug transporter function which can then affect the effectiveness of drugs (Breedveld et al., 2007).

Collectively these studies demonstrate how inflammation-induced cytokines and TLRs are involved in regulating the expression of drug transporters in various tissues; subsequently altering intracellular and plasma drug concentrations, thereby affecting drug pharmacokinetics and efficacy. Inflammation mediated changes in drug transporter expression are however mostly based on animal models and cell lines (Cressman et al., 2012; Saib and Delavenne, 2021), thereby warranting the need for comparative *in-vivo* human studies. Future studies should also elucidate how BV, HC and STIs-induced genital inflammation contribute to drug transporter expression and function, subsequently, predisposing women to HIV infections, even during PrEP intake. Therefore, additional studies are needed to understand the interplay between inflammation and drug transporter expression, especially in sites highly susceptible to HIV such as the FGT and blood. Findings from such studies would provide a better understanding of how the presence of systemic and genital inflammation may alter drug transporters subsequently affecting ARV pharmacokinetics. Further elucidation of these factors either individually or collectively will aid in understanding disparities in PrEP efficacies observed in PrEP trials. This is especially important in highly susceptible groups such as African women from HIV endemic settings where PrEP is advocated as the standard of care for HIV prevention.

**FIGURE 2:**
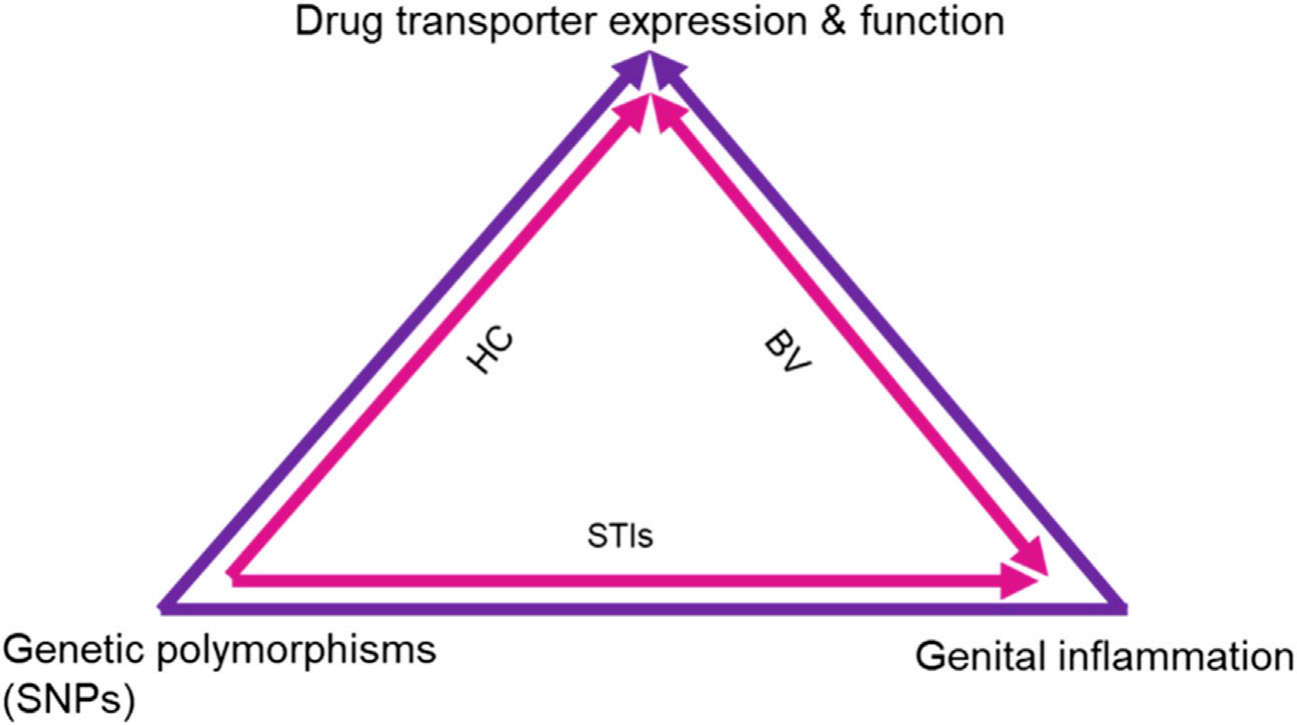
**Proposed mechanism of effects on drug transporter expression and function.** The schematic shows the intersection of different biological factors and SNPs in drug transporter genes that affect drug transporter expression in the FGT, renal system and blood, subsequently affecting PrEP efficacy. Genital inflammation and SNPs are known to directly affect drug transporter expression and functionality, while the combined use of HCs and ARVs also affects drug transporter expression and function. Additionally, the presence of STIs and BV are shown to contribute to genital inflammation which in turn affects drug transporter expression and function. HC, Hormonal contraceptives; BV, Bacterial vaginosis; STIs, Sexually transmitted infections.

Together these studies show evidence that there may be an inextricable link between the expression and function of drug transporters with genetic polymorphisms, TLRs, pH and genital inflammation, which is further influenced by the presence of BV, STIs and HCs (Figure 2). Subsequently these factors may significantly affect drug concentrations and potentially drug efficacies.

## 8 Conclusion

The current review provides evidence that the FGT, renal system and blood are subject to a variety of host biological factors that may undermine PrEP efficacy by affecting drug transporter expression levels and function. These afore-mentioned studies show how drug transporters are increasingly recognised as key determinants in drug pharmacokinetics and response. However, their contributions to the inconsistent efficacies seen in PrEP clinical studies in African women from regions with high HIV infection rates such as South Africa, have not been elucidated. Characterising the expression level of drug transporters in the blood and FGT from a vulnerable population will better define the biological factors underlying compartment variation in drug exposure during oral PrEP in at-risk African women. In turn, we may be able to better understand why African women remain susceptible to HIV despite PrEP interventions. Additionally, findings from such studies will shed an important light on how the genetics and the biology of the mucosal environment may play a pivotal role in modifying drug transporter expression, subsequently modulating HIV risk. Understanding these data may also aid in the development of more effective, safe and optimal delivery systems that facilitate consistent effective dosage and usage of appropriate PrEP drugs.
